# Structural and biochemical characterization of *Plasmodium falciparum* Hsp70‐x reveals functional versatility of its C‐terminal EEVN motif

**DOI:** 10.1002/prot.25600

**Published:** 2018-09-29

**Authors:** Blessing Mabate, Tawanda Zininga, Lebogang Ramatsui, Stanley Makumire, Ikechukwu Achilonu, Heini W. Dirr, Addmore Shonhai

**Affiliations:** ^1^ Department of Biochemistry University of Venda South Africa; ^2^ Protein Structure‐Function Research Unit School of Molecular & Cell Biology, University of the Witwatersrand Johannesburg South Africa

**Keywords:** asparagine repeat rich peptide, chaperone, EEVN, heat shock proteins, human hop, PfHsp70‐x

## Abstract

*Plasmodium falciparum*, the main agent of malaria expresses six members of the heat shock protein 70 (Hsp70) family. Hsp70s serve as protein folding facilitators in the cell. Amongst the six Hsp70 species that *P. falciparum* expresses, Hsp70‐x (PfHsp70‐x), is partially exported to the host red blood cell where it is implicated in host cell remodeling. Nearly 500 proteins of parasitic origin are exported to the parasite‐infected red blood cell (RBC) along with PfHsp70‐x. The role of PfHsp70‐x in the infected human RBC remains largely unclear. One of the defining features of PfHsp70‐x is the presence of EEVN residues at its C‐terminus. In this regard, PfHsp70‐x resembles canonical eukaryotic cytosol‐localized Hsp70s which possess EEVD residues at their C‐termini in place of the EEVN residues associated with PfHsp70‐x. The EEVD residues of eukaryotic Hsp70s facilitate their interaction with co‐chaperones. Characterization of the role of the EEVN residues of PfHsp70‐x could provide insights into the function of this protein. In the current study, we expressed and purified recombinant PfHsp70‐x (full length) and its EEVN minus form (PfHsp70‐x_T_). We then conducted structure‐ function assays towards establishing the role of the EEVN motif of PfHsp70‐x. Our findings suggest that the EEVN residues of PfHsp70‐x are important for its ATPase activity and chaperone function. Furthermore, the EEVN residues are crucial for the direct interaction between PfHsp70‐x and human Hsp70‐Hsp90 organizing protein (hHop) in vitro. Hop facilitates functional cooperation between Hsp70 and Hsp90. However, it remains to be established if PfHsp70‐x and hHsp90 cooperate in vivo.

## 1 INTRODUCTION

Malaria persists as a major cause of morbidity and deaths around the world, with the latest data showing that it accounts for ~450 000 deaths annually.^1^ *Plasmodium falciparum* is the most virulent of all the species that cause malaria. It is during the development of the parasite at the blood stage that clinical malaria manifests. In addition, the development of clinical malaria is associated with periodic fever conditions. As part of its response to physiological changes, the malaria parasite is thought to employ its arsenal of heat shock proteins (Hsps). Hsps are molecular chaperones that assist in folding of other proteins. Hsp70 constitute one of the main molecular chaperones of the cell. Structurally, Hsp70 is composed of a conserved N‐terminal (ATPase) domain and a less conserved C‐terminal substrate binding domain (SBD). Most cytosolic Hsp70s, possess an EEVD motif positioned at the end of the SBD. The EEVD motif is thought to play a role in the interaction of Hsp70 with its cochaperones such as members of the Hsp40 family and another distinct co‐chaperone, Hsp70‐Hsp90 organizing protein (Hop).^2^ Notably, *E. coli* Hsp70 (DnaK) possesses EEVKDKK residues at its C‐terminus in comparison with cytosolic Hsp70s of human and plasmodial origin.^3^

Hsp40 co‐chaperones stimulate the otherwise low basal ATPase activity of Hsp70 chaperones.^4^ In addition, Hsp40s bind substrates which they pass on to Hsp70 thus regulating substrate specificity of the latter.^4^ Hop is a co‐chaperone that serves as a module that brings Hsp70 in functional complex with another chaperone, Hsp90.^5^ This association facilitates the partial folding of some substrates by Hsp70 and whose final folding requires Hsp90.^6^

*P. falciparum* expresses 6 Hsp70s of which, PfHsp70‐x (PlasmoDB: Accession number PF3D7_0831700), is exported to the host red blood cell (RBC) cytosol.^7^, ^8^ Hsp70‐x homologues only occur in *P. falciparum* and the chimpanzee malaria agent, *P. reichinowi.* Thus, the exclusive presence of Hsp70‐x in the most virulent plasmodial species suggests a possible role of this protein in malaria pathogenicity.^9^, ^10^ PfHsp70‐x possesses an N‐terminal signal peptide of 24 amino acids which potentially directs the protein to the endoplasmic reticulum (ER).^7^, ^8^ The absence of the ER retention sequence suggests that the chaperone passes through the ER before being exported.^7^ Interestingly, PfHsp70‐x does not contain the plasmodium export element (PEXEL) (pentapeptide) motif of which most RBC exported parasite proteins possess.^11^

PfHsp70‐x is reportedly secreted into the parasitophorous vacuole (PV) and some of it is exported into the host RBC.^8^ In addition, PfHsp70‐x is thought to occur in the Maurer's clefts as it colocalizes with MAHRP1, a Maurer's cleft marker which suggests that the chaperone may be involved in parasite protein sorting and export.^12^ However, other studies have reported that PfHsp70‐x does not occur in the Maurer's clefts but instead is located in distinct subcellular structures termed “J‐dots”.^13^, ^14^ A study by Daniyan and colleagues^15^ confirmed that a plasmodial Hsp40, PFA0660w, directly binds to PfHsp70‐x. This strongly implies that PfHsp70‐x could play a role in chaperoning proteins of parasitic origin that are exported to the RBC. Although, PfHsp70‐x is not essential, two recent independent studies^9^, ^10^ suggested that RBCs infected by parasites lacking the *PfHsp70‐x* gene demonstrated reduced cyto‐adherence, thus implicating PfHsp70‐x in infectivity. In addition, it was further suggested by Charnaud et al.^9^ that PfHsp70‐x may play an important role in host immune evasion.

Human chaperones have since been reported to associate with some proteins of parasitic origin that are exported to the infected host RBC.^13^ This association is important in the development of malaria pathogenicity and infectivity of *P. falciparum* 9, 15 as some of the protein complexes are responsible for modifying the infected RBC making it cyto‐adherent.^16^ Detergent solubilization experiments have shown that human chaperones (Hsp90, Hsp70, and Hop) are present in the uninfected RBCs and occur in soluble form.^17^ However, in infected RBCs, the human chaperones are detected in knob enriched fractions as insoluble complexes that are cross‐linked to virulence proteins suggesting that the host chaperone system is functionally diverted during knob formation.^17^ It is known that Hsp70, Hop and Hsp90 are able to form a complex (Hsp70‐Hop‐Hsp90) which is important for the folding of some proteins.^18^, ^19^ The presence of PfHsp70‐x in the infected RBCs presents a possibility that this protein may functionally cooperate with human Hsp90 (hHsp90) via human Hop (hHop) as the parasite does not export its own Hop or Hsp90 proteins.^5^ In place of the C‐terminal EEVD motif normally present in cytosolic Hsp70, PfHsp70‐x possesses an EEVN motif.^2^ It is possible that the presence of the EEVN motif may facilitate interaction of PfHsp70‐x with cochaperones, amongst them, hHop. The possible occurrence of a hHop‐mediated partnership between PfHsp70‐x and PfHsp90 could constitute a novel protein folding pathway that is managed through cooperation of molecular chaperones from two distinct species. Therefore, in the current study we investigated the possible interaction of PfHsp70‐x with human Hop. Furthermore, it is known that *P. falciparum* proteins are rich in asparagine repeat regions as compared to human proteins.^20^ For this reason, we investigated the substrate binding preferences of PfHsp70‐x.

## 2 MATERIALS AND METHODS

### 2.1 Materials

The chemical reagents used in the study were generally sourced from the following suppliers; Merck Chemicals (Darmstadt, Germany), Thermo Scientific (IL, USA), Zymo Research (USA), Melford (Suffolk, UK), and Sigma–Aldrich (USA). The Nickel NTA resin was purchased from Thermo Scientific (USA), while the ECL kit was bought from (GE Healthcare, Germany). The α‐His antibody that was used in the study was purchased from (Thermo Scientific, USA). The α‐PfHsp70‐x antibody produced in rabbit^8^ was kindly provided by Prof. Jude Pryzboski, Philipps‐Marburg University, Germany. The α‐DnaK antibody was secured from Stressgen, USA.

### 2.2 Generation of expression constructs

Codon harmonized forms of DNA encoding hHop, both the full length PfHsp70‐x (PlasmoDB accession # PF3D7_0831700) and its c‐terminal truncated version, with a missing EEVN motif (PfHsp70‐x_T_), were synthesized by GenScript (USA). The DNA segments encoding the respective proteins were subsequently cloned onto the pQE30 (Qiagen) vector using *Bam*HI and *Hind*III restriction sites in frame with an N‐terminal histidine tag. The integrity of the resulting constructs was verified by restriction analysis and DNA sequencing.

### 2.3 Expression and purification of recombinant proteins

Constructs expressing PfHsp70‐x (pQE30/PfHsp70‐x) and PfHsp70‐x_T_ (pQE30/PfHsp70‐x_T_), respectively, were used for the expression of recombinant PfHsp70‐x and its truncated version, PfHsp70‐x_T_. The recombinant proteins were expressed in *E. coli* XL1 Blue cells, following previously described protocols.^19^, ^21^ Similarly, the construct expressing hHop (pQE30/hHop) was used for the expression of hHop. The recombinant proteins were expressed in frame with N‐terminally attached polyhistidine tags which facilitated purification using affinity chromatography as previously described.^19^, ^21^

### 2.4 Analysis of the secondary structure organization and the heat stability of PfHsp70‐x

The secondary structure of recombinant PfHsp70‐x was analyzed using Far‐UV circular dichroism (CD) as previously described.^22^ Briefly, 0.2 μM PfHsp70‐x recombinant protein was suspended in 20 mM Tris‐HCl pH 7.4, 100 mM sodium fluoride buffer. The analysis was conducted on a J‐1500 CD spectrometer (JASCO Ltd, UK) equipped with a Peltier temperature controller at 19°C, using a 0.1 cm path‐length quartz cuvette (Hellma, Germany). Spectra were averaged for at least 15 scans taking into account the baseline scan (buffer in which recombinant protein was excluded). The data were analyzed and de‐convoluted to α‐helix, β‐sheet, β‐turn and unordered regions using the CDSSTR program of the Dichroweb online server.^23^ In addition, the thermal stability of the recombinant protein was determined by fixing the wavelength at 222 nm and subjecting the protein to stepwise increase in temperature from 19°C to 99°C.^22^

### 2.5 Investigation of the nucleotide dependent conformational changes of PfHsp70‐x using tryptophan fluorescence based analysis

Nucleotide dependent conformational perturbations of PfHsp70‐x were investigated by tryptophan fluorescence as previously described.^22^ Briefly, recombinant protein (0.45 μg mL^−1^) was incubated in the absence and presence of 5 mM nucleotide ATP/ADP in assay buffer A (25 mM HEPES‐KOH pH 7.5, 100 mM KCl, 10 mM MgOAc) for 30 minutes at 20°C. Fluorescence was measured between 320 and 450 nm after initial excitation at 295 nm using the JASCO FP‐6300 spectrofluorometer. Relative fluorescence was calculated as the average value obtained from at least 7 spectrum scans less the baseline (buffer with or without nucleotides in the absence of protein) reading.

### 2.6 Determination of the nucleotide binding affinity of PfHsp70‐x

The nucleotide binding assay was conducted at 25°C using a BioRad ProteOn XPR36 surface plasmon resonance (SPR) as previously described.^22^ The assay buffer was constituted of filter sterilized and degassed PBS‐Tween [(10 mM Na_2_HPO_4_, 1.46 mM KH_2_PO_4,_ 137 mM NaCl, 3 mM KCl, 0.005% [v/v] Tween 20 and 20 mM EDTA; pH 7.4)]. PfHsp70‐x (1 μg mL^−1^) and its truncated version, PfHsp70‐x_T_ (1 μg mL^−1^) were immobilized onto respective ProteOn HTE sensor chips as ligands. At these concentrations, we achieved 190 response units (RU) for PfHsp70‐x and 195 RU for PfHsp70‐x_T_ per immobilization surface. Varying concentrations (1.25 nM, 2.50 nM, 5 nM 10 nM and 20 nM) of the analyte (ATP) were injected at a flow rate of 100 μL min^−1^ in each horizontal channel and association and dissociation was allowed to occur and monitored for 10 minutes. Steady state equilibrium constant data was processed and analyzed using ProteOn Manager software version 3.1.0.6.

### 2.7 ATPase assay

The ATPase activity of PfHsp70‐x was determined by quantifying the amount of released inorganic phosphate based on a previously described approach.^24^ Briefly, 0.4 μM PfHsp70‐x was incubated for 5 minutes in buffer HKM (10 mM HEPES‐KOH pH 7.5, 100 mM KCl, 2 mM MgCl_2_, 0.5 mM DTT). The reaction was started by addition of ATP and samples were collected after 30 minutes. Kinetic plots for the ATPase activities of PfHsp70‐x and PfHsp70‐x_T_ were determined by generating Michaelis‐Menten plots. At least three independent batches of recombinant protein were used in separate experiments. Statistical analysis was conducted using GraphPad prism 6.05.

### 2.8 Protein aggregation suppression assay

The capability of PfHsp70‐x and PfHsp70‐x_T_ to suppress heat‐induced aggregation of luciferase obtained from *Photinus pyralis* (Sigma–Aldrich, USA) was investigated. The experiment was conducted as previously described^22^ with minor modifications. The assay was conducted by suspending 0.6 μM luciferase and 0.2 μM of PfHsp70‐x/PfHsp70‐x_T_ in assay buffer HMN (25 mM HEPES‐KOH, pH 7.5, 5 mM Mg[OA_C_,]_2,_ 5 mM, NaOA_C_, 50 mM KCl, 5 mM β‐mercaptoethanol) and exposed to 48°C for 90 minutes. The assay was repeated in the presence of varying levels (0 μM, 0.3 μM, 0.6 μM, and 1.2 μM) of PfHsp70‐x/PfHsp70‐x_T_. To determine the effect of nucleotides on the chaperone function of PfHsp70‐x/PfHsp70‐x_T_, the assay was repeated in the presence of 5 mM ATP/ADP and equimolar concentrations (0.6 μM) of luciferase and chaperone, respectively. The resulting aggregation of luciferase was monitored at 340 nm using SpectraMax M3 spectrometer (Molecular Devices, USA). In place of PfHsp70‐x/PfHsp70‐x_T_, bovine serum albumin (BSA) was used as a non‐chaperone control.

The chaperone function of PfHsp70‐x and PfHsp70‐x_T_ was further monitored using a second aggregation prone model protein, malate dehydrogenase (MDH) from porcine heart (Sigma–Aldrich, USA) as previously described.^21^, ^22^, ^24^, ^25^ The MDH aggregation assay was conducted under the same experimental conditions as described for luciferase above. The data generated from the aggregation assays were analyzed using GaphPad Prism 6.05 software.

### 2.9 Determination of peptide substrate binding kinetics

Association of PfHsp70‐x/PfHsp70‐x_T_ with synthetic peptides that represent typical Hsp70 substrates^26^ was investigated using surface plasmon resonance (SPR) analysis as previously described.^22^, ^24^ Briefly, PfHsp70‐x and PfHsp70‐x_T_ were immobilized onto respective ProteOn GLC sensor chips as ligands. PfHsp70‐x, PfHsp70‐x_T_ (as ligands) were immobilized at concentrations of 0.5 and 1 μg mL^−1^. At these concentrations we achieved 190 response units (RU) for PfHsp70‐x, and 193 RU for PfHsp70‐x_T_ per immobilization surface. The assay was conducted at 25°C. Filter sterilized and degassed PBS‐Tween was used as the running buffer. As analytes, aliquots of peptides, representing typical Hsp70 substrates (NRLLTG, NRNNTG, ALLLMYRR, ANNNMYRR, GFRVVLMYRF, GFRNNNMYRF) were prepared at final concentration of 1.25, 2.50, 5, 10, and 20 nM and were injected at 50 μL min^−1^ in each horizontal channel. Association was allowed for 2 minutes, dissociation was monitored for 10 minutes. The assay was repeated in the presence of nucleotides 2 mM ATP/ADP. The final data were determined by substracting the baseline RU (buffer containing ATP/ADP without analyte peptide). The association rate constant, dissociation rate constant and equilibrium constant data were analyzed using ProteOn Manager software version 3.1.0.6. by concatenating the responses of the analyte at variable concentrations.

### 2.10 Determination of the direct interaction between PfHsp70‐x and hHop

Recombinant forms of hHop, control (bovine serum albumin), PfHsp70‐x (positive control) were immobilized onto the nitrocellulose membrane using Bio‐Dot SF apparatus as previously described 22. The proteins were immobilized at final amounts of 2, 4, and 8 μg each respectively per lane. The membrane was blocked with 5% (w/v) fat‐free milk in TBST (50 mM Tris‐HCl pH 7.5, 150 mM NaCl, 0.1% [v/v] Tween 20) and overlaid with either PfHsp70‐x/PfHsp70‐x_T_ (10 μg mL^−1^) in the absence and presence of 5 mM ATP/ADP and incubated overnight at 4°C, followed by three washes with TBST for 15 minutes. The nucleotide concentrations were maintained in the subsequent steps of the protocol. Protein was detected by Western blot technique using rabbit raised α‐PfHsp70‐x [1:2000] (Genscript, USA) as primary antibodies and goat raised α‐rabbit IgG secondary HRP‐conjugated antibody [1:4000] (Sigma–Aldrich, USA). Imaging of the protein bands was conducted using the ECL kit as per manufacturer's instructions. The images were captured using ChemiDoc imaging system (Bio‐Rad, USA).

The direct association of hHop with PfHsp70‐x was further confirmed using an enzyme linked immunosorbent assay (ELISA) as previously described^27^ with minor modifications. Briefly, recombinant PfHsp70‐x/PfHsp70‐x_T_ proteins and control BSA (5 μg/mL) were added to wells overnight in 50 mM NaHCO_3_, pH 9.3 at 4°C. The wells were washed at least three times with TBST, between each subsequent step. After blocking with 1% BSA in TBST, recombinant PfHop protein (analyte) was added in serial dilutions (0.137–300 nM) in the presence or absence of nucleotides (5 mM ATP/ADP) for 2 hours at 25°C. Then the wells were incubated with α‐PfHop primary antibody for 1 hour. Next, primary antibody was incubated with HRP conjugated goat raised anti‐rabbit IgG for 1 hour. HRP development was assayed with TMB substrate solution (Bio‐Scientific, USA) and monitored at 370 nm using a SpectraMax M3 microplate reader (Molecular Devices, USA) every 5 minutes for 30 minutes.

The direct association between PfHsp70‐x and hHop was further explored using SPR analysis. The assay was conducted at 25°C using filter sterilized and degassed PBS‐Tween running buffer [(8 mM Na_2_HPO_4_, 1.46 mM KH_2_PO_4_, 137 mM NaCl, 3 mM KCl, 0.005% [v/v] Tween 20 and 20 mM EDTA; pH 7.4)] as previously described.^22^, ^24^ The recombinant PfHsp70‐x /PfHsp70‐x_T_ (1 μg mL^−1^) and BSA (as control) were immobilized onto respective ProteOn GLC sensor chips as ligands. As analyte, the recombinant hHop at varying concentrations (125, 250, 500, 1 000, and 2 000 nM) was passed over the immobilized proteins. The analytes were injected at flow rate of 50 μL min^−1^ and association and dissociation occurred in a period of 10 minutes. The assay was then repeated in the presence of 5 mM ADP/ATP. The association rate constant, dissociation rate constant, and equilibrium constant data were processed and analyzed using ProteOn Manager software version 3.1.0.6 involving concatenating the responses of the analyte at variable concentrations.

## 3 RESULTS

### 3.1 The C‐terminal EEVN motif of PfHsp70‐x regulates the chaperone's conformation

**Figure 1:**
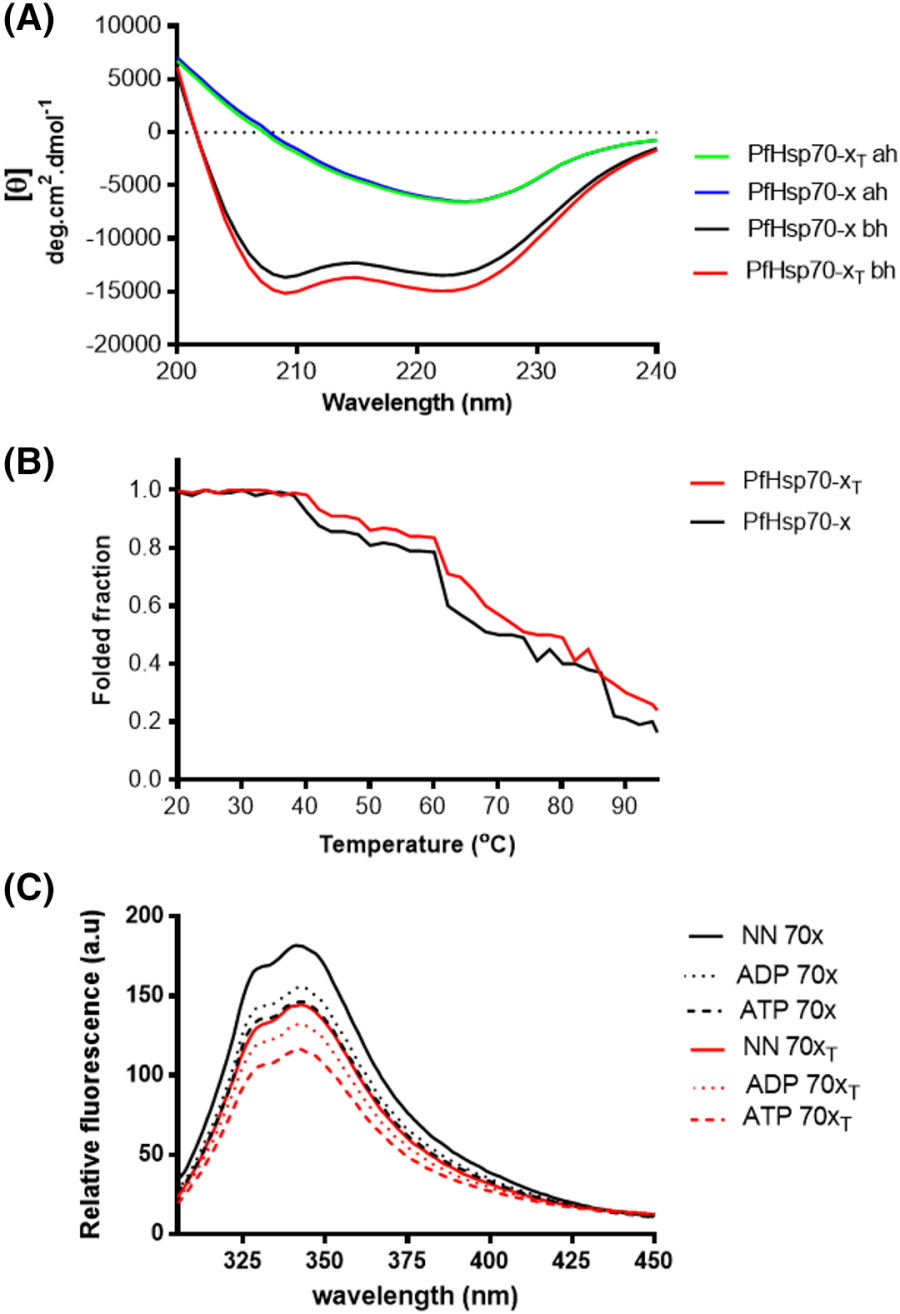
PfHsp70‐x is predominantly alpha‐helical and thermally stable. A, The UV spectra of recombinant full length and truncated PfHsp70‐x protein. The CD spectrum of PfHsp70‐x was presented as molar ellipticity (θ), before heating (bh) and after heating the protein at 90°C (ah). Spectra were averaged for least 15 scans after subtraction of baseline scan (buffer in which PfHsp70‐x protein was excluded). B, The UV spectra of the folded fractions of both full length and truncated PfHsp70‐x under varying temperature conditions. Readings were taken at 222 nm. C, Tryptophan fluorescence signals obtained for both full length and EEVN minus protein forms in the absence of nucleotide; and tryptophan fluorescence signals obtained for both full length and EEVN minus protein forms in the presence of 5 μM ATP/ADP. Relative fluorescence was calculated as the average value obtained from at least seven spectrum scans less the baseline (buffer with or without nucleotides in the absence of protein) reading [Color figure can be viewed at http://wileyonlinelibrary.com]

The effect of heat stress on the secondary structure of PfHsp70‐x was investigated by conducting CD spectrophotometric analysis. The purified recombinant PfHsp70‐x (full length) and its truncated version (Supporting Information Figure 1) were incubated at 19°C. Changes in CD ellipticity were recorded for wavelengths spanning between 200 nm and 240 nm (Figure 1A). The assay was repeated under varying temperature conditions (19°C ‐ 95°C) (Figure 1A). The far‐UV spectra exhibited two negative peaks at 208 and 222 nm, respectively, representing the helical character of the proteins. The deconvolution of the CD spectra revealed that the full‐length PfHsp70‐x is predominantly composed of α‐helices (51%), followed by β‐strands (23%), β‐turns (14%) and unordered (12%). The removal of the EEVN motif resulted in a product without a significant shift in secondary structural conformation compared to the wild type form of the protein (Figure 1A).

Sustained heat stress led to loss of protein‐fold for both the full length and the truncated (EEVN minus) forms of PfHsp70‐x as observed using CD spectrometry at wavelengths 200 nm to 240 nm (Figure 1A). A close analysis of the effects of heat stress was further evaluated at a fixed wavelength (221 nm) under varying temperature conditions (19°C ‐ 95°C) (Figure 1B). Generally, both wild type protein and the EEVN deletion mutant were stable at temperatures from 19°C to 40°C. However, upon increasing heat stress, the proteins unfolded in two distinct gradients [(40°C to 60°C) and (60°C to 90°C)]. This suggests that both forms of the protein were generally denatured in two distinct phases upon being exposed to temperatures from 40°C and 90°C. The removal of the EEVN motif did not appear to adversely influence the‐fold of the protein in comparison to the wild type form. In fact, the EEVN minus form of the protein was marginally more stable that the wild type form at temperatures ranging from 40°C and 90°C. Overall, as we previously observed for the cytosolic isoform, PfHsp70‐1,^22^, ^28^ at least 50% of PfHsp70‐x appeared stable at temperatures up to 70°C (Figure 1B). This further suggests that the malaria parasite is endowed with Hsp70s that are generally resilient to stressful conditions.

In a separate experiment, the tertiary structure of PfHsp70‐x and its truncated version were evaluated using tryptophan based fluorescence analysis of the protein in the absence of nucleotides and presence of nucleotides (Figure 1C), respectively. We observed a reduction in emission maximum peak on the EEVN truncated version of the protein both in the absence and presence of nucleotides (ATP/ADP). This finding mirrors a similar trend to observations we made with respect to the effect of heat stress on the secondary structures of both the full length and the EEVN minus forms of the PfHsp70‐x. Altogether, our findings suggest that the EEVN motif does contribute to the conformational integrity of PfHsp70‐x. While, the introduction of ADP did not appear to shift the emission peak, the presence of ATP, reduced the emission peak, suggesting a change in the conformations of both the full length and EEVN deletion forms of PfHsp70‐x (Figure 1C). This is in agreement with a previous observation we made that the parasite cytosolic Hsp70 homologue of PfHsp70‐x, PfHsp70‐1 assumes a similar conformation in the absence of nucleotide as in the presence of ADP.^22^ On the other hand, ATP modulated the conformation of PfHsp70‐x (Figure 1C) in a similar fashion to the observation we previously made for the cytosolic isoform, PfHsp70‐1.^21^, ^22^

### 3.2 The EEVN motif of PfHsp70‐x is important for ATP binding

**Figure 2:**
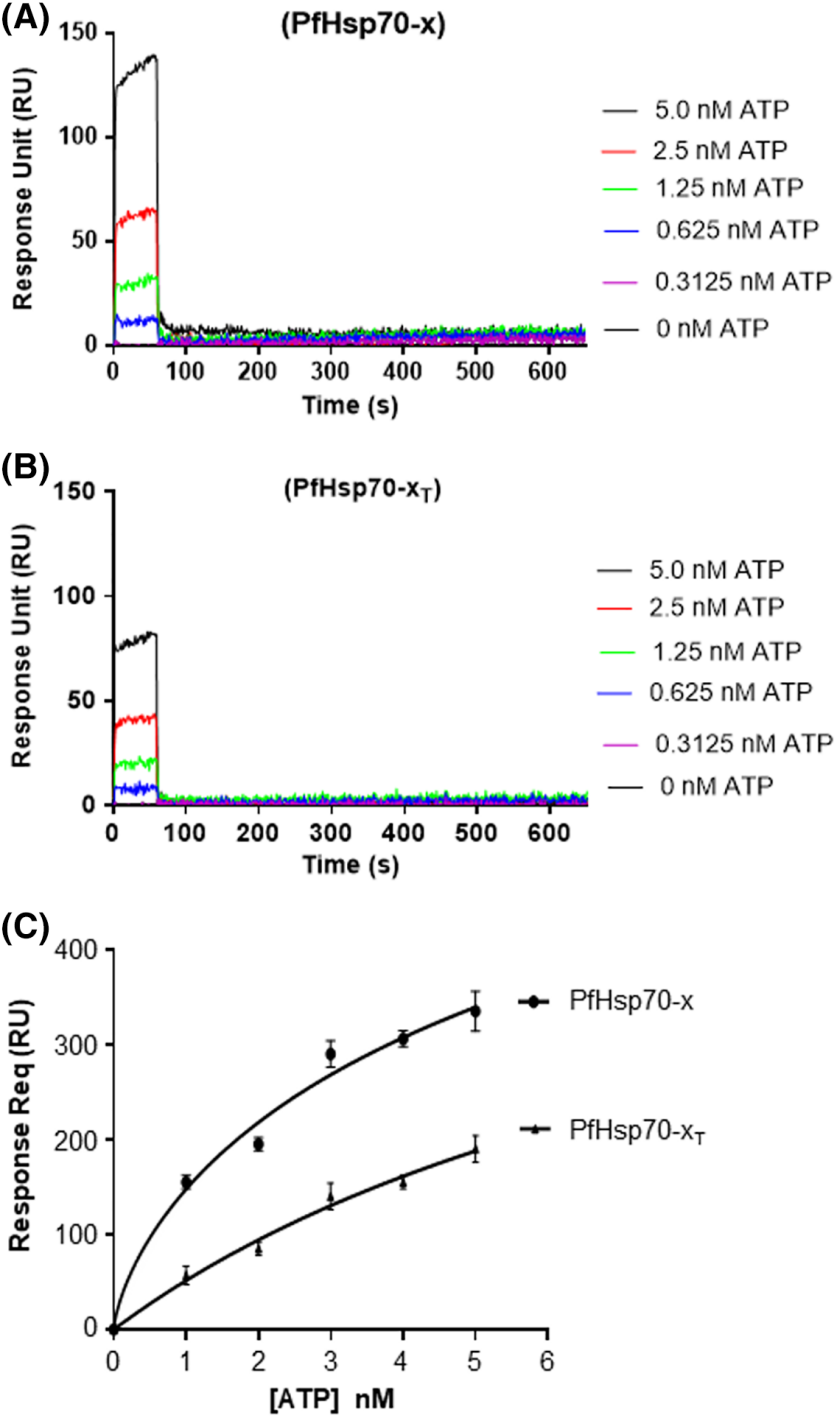
Equilibrium analysis of ATP‐binding by PfHsp70‐x and PfHsp70‐x_T._ The representative sensorgrams generated from the SPR analysis for PfHsp70‐x‐ATP association (A) and PfHsp70‐x_T_‐ATP association (B) are shown. The data represent the equilibrium analysis of ATP binding constants for both proteins (C). The analysis was conducted in the presence of variable levels of ATP. The data represent three independently conducted assays run in duplicates

PfHsp70‐x and PfHsp70‐x_T_ ATP binding affinities were determined using SPR (Figure 2). The equilibrium analysis for the binding affinities were determined at steady state with the respective forms of PfHsp70‐x as ligand under variable ATP levels and respective analyte each time. No signal was observed when BSA, a non‐chaperone control was used as ligand. PfHsp70‐x_T_ exhibited a hundred‐fold lower ATP binding affinity compared to PfHsp70‐x (Table 1). This suggests that the removal of the EEVN motif had adverse effects on the ATP binding capability of PfHsp70‐x.

**Table 1:** Comparative affinities of PfHsp70‐x and PfHsp70‐x_T_ for ATP binding

Ligand	ATP analyte
	*K*_*D*_ in μM (± std deviations)
PfHsp70‐x	0.11 (± 0.03)
PfHsp70‐x_T_	11.2 (± 1.76)

Legend: Standard deviation shown were generated from three independent assays.

### 3.3 The EEVN motif of PfHsp70‐x regulates the ATPase activity of the protein

**Figure 3:**
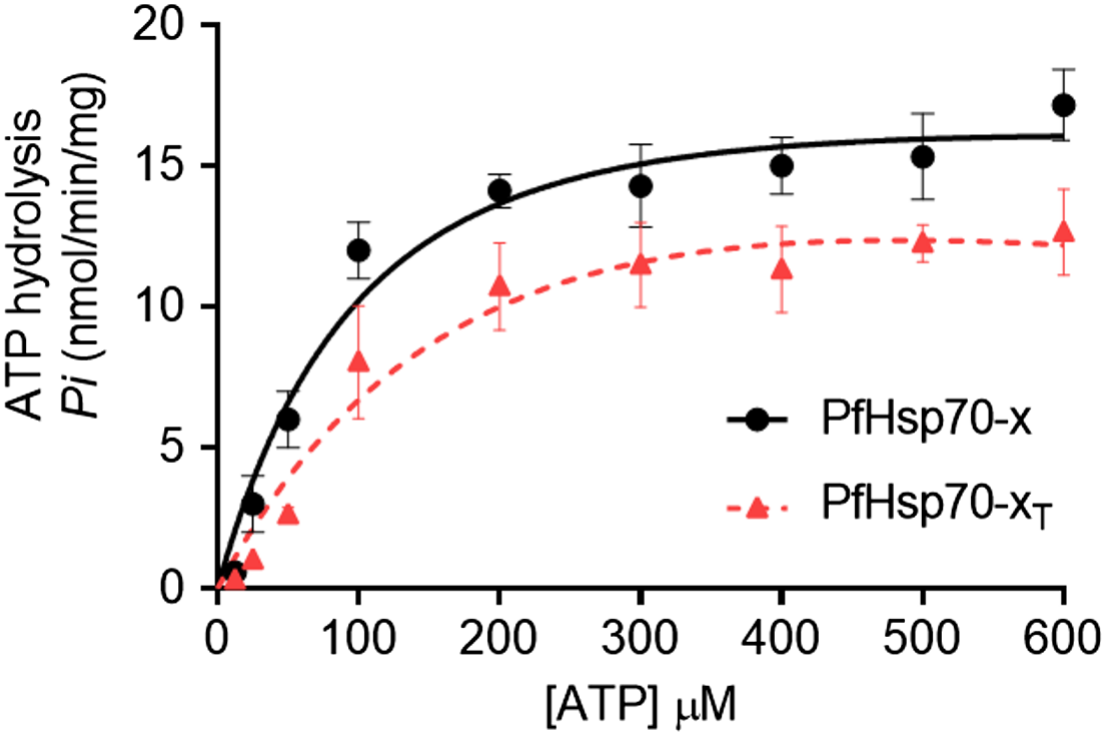
Analysis of the basal ATPase activity of PfHsp70‐x. The inorganic phosphate released was monitored by direct colourimetry at 595 nm wavelength. The curves represent basal ATPase activities of PfHsp70‐x and PfHsp70‐x_T_, respectively expressed as mean (±) SD. The assay was carried out in duplicate. At least three assays were conducted using fresh protein batches each time [Color figure can be viewed at http://wileyonlinelibrary.com]

The basal ATPase activity of PfHsp70‐x and PfHsp70‐x_T_ were determined using a colorimetric assay.^24^ The assay was conducted under variable concentrations (0‐600 μM) of ATP. The Michaelis‐Menten plots for PfHsp70‐x and PfHsp70‐x_T_ were determined using at least three independent batches of the recombinant proteins (Figure 3). Both PfHsp70‐x and PfHsp70‐x_T_ exhibited intrinsic ATPase activity within comparable range to the previously reported activity of PfHsp70‐1 (Figure 3 ^24^). However, the *K*_m_ value obtained for PfHsp70‐x_T_ was higher in magnitude compared to that obtained using the full‐length protein. This further shows that removal of the EEVN motif resulted in a protein with reduced affinity for ATP (Table 2).

**Table 2:** ATPase kinetics of PfHsp70‐x

Protein	*V*_max_ (nmol/min/mg)	*K*_m_ (μM)	Reference
PfHsp70‐x	14.70 (± 0.42)	21.4 (± 3.40)	This study
PfHsp70‐x_T_	12.70 (± 0.52)	50.0 (± 8.80)	This study
PfHsp70‐x	17.20 (± 0.61)	393.0 (± 93.00)	(Cockburn et al 2014**)** 33
PfHsp70‐x	12.80 (± 0.13)	ND	(Daniyan et al 2016)^15^

Table legends: ND, not determined. SDs shown represent at least three independent assessment made using separate protein purification batches.

### 3.4 PfHsp70‐x and PfHsp70‐x_T_ suppressed heat‐induced aggregation of luciferase and malate dehydrogenase (MDH)

**Figure 4:**
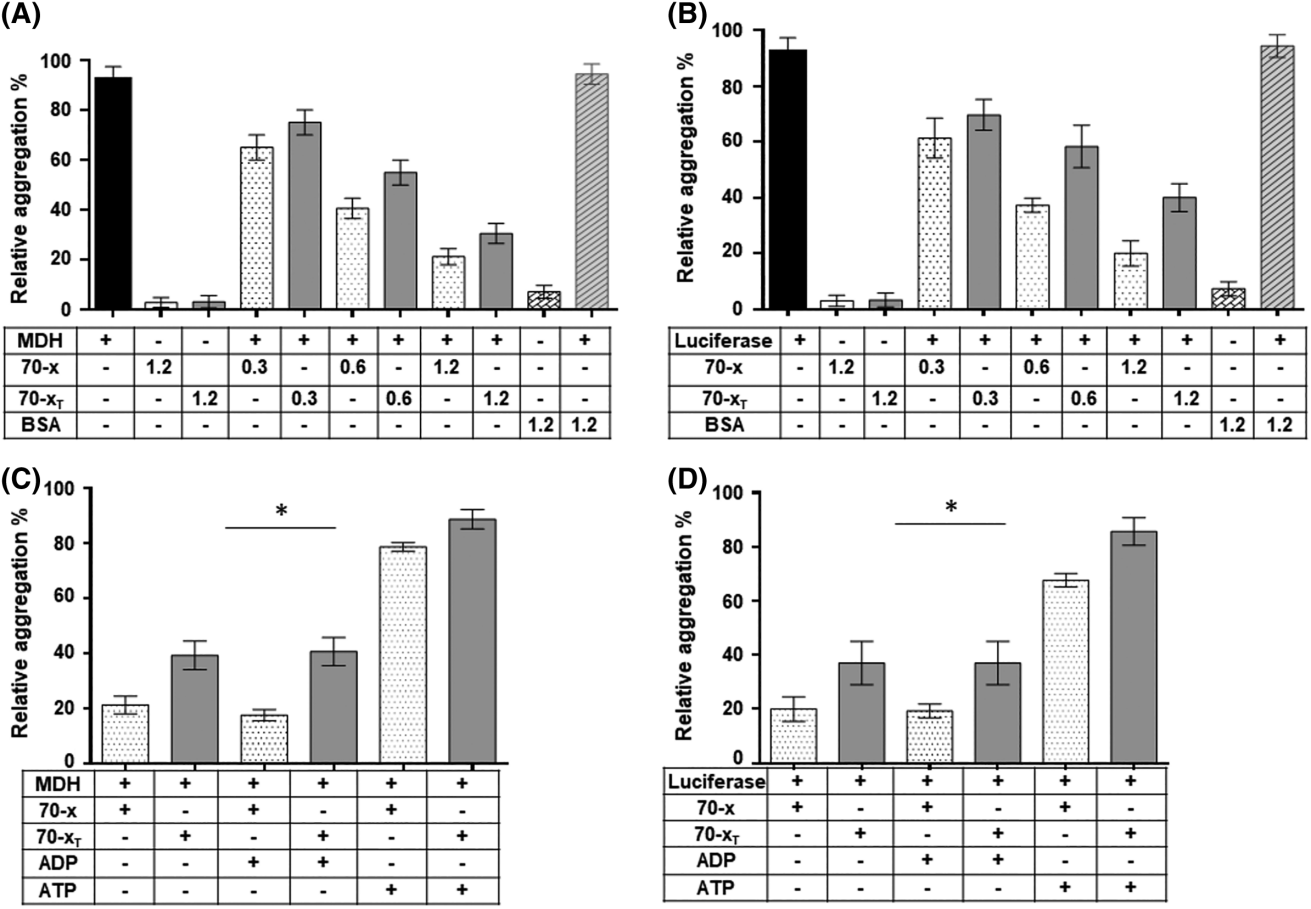
PfHsp70‐x suppresses heat‐induced aggregation of luciferase and malate dehydrogenase. The heat‐induced aggregation of model substrates (luciferase and MDH) was monitored in vitro at 48 °C using a J‐1500 CD spectrometer (JASCO Ltd, UK) at wavelength of 340 nm. The heat‐induced aggregation of 0.6 μM MDH (A) represented by relative percentages of turbidity was measured using a UV–vis spectrophotomer at 340 nM (A) and that of 0.6 μM luciferase (B), respectively. The assays were conducted in the presence of variable levels (of PfHsp70‐x/PfHsp70‐x_T_; 1.2 μM BSA) was used as a non‐chaperone control. The heat‐induced aggregation of 0.6 μM PfHsp70‐x/PfHsp70‐x_T_ and 0.6 μM MDH (**C**), 0.6 μM luciferase (**D**) was monitored either in the absence of nucleotide or presence of 5 μM ADP/ATP at equimolar levels of substrate/chaperone ratio. The assays were repeated at least three times using independently purified recombinant protein batches. The statistical analysis was conducted using one way anova. *P* < .005* represent statistically significant differences in activities noted under the variable experimental conditions

The ability of PfHsp70‐x to suppress heat‐induced aggregation of model substrates (luciferase and MDH) was investigated at 48°C by observing the change in turbidity of the respective reaction mixtures at 340 nm. First, it was important to establish that recombinant proteins (PfHsp70‐x and PfHsp70‐x_T_) would not aggregate at 48°C (Figure 4A,B). This is in line with our CD data that showed that both PfHsp70‐x and PfHsp70‐x_T_ are generally stable at high temperatures as up to 50% of the proteins remained folded at temperatures as high as 70°C (Figure 1B). As expected, luciferase and MDH aggregated at 48°C in the absence of chaperones or in the presence of the non‐chaperone control, BSA. The degree of luciferase/MDH aggregation (based on optical density) observed was arbitrarily set as 100% (Figure 4A,B). Both PfHsp70‐x and PfHsp70‐x_T_ displayed capability to suppress the aggregation of luciferase/MDH in a concentration‐dependent fashion (Figure 4A,B). Interestingly, compared to the full length protein, PfHsp70‐x_T_ was generally less effective at suppressing the heat‐induced aggregation of either luciferase or MDH (*P* < .001). This suggests that removal of the EEVN motif impacted adversely on the chaperone activity of PfHsp70‐x. The effect of nucleotides on the capability of PfHsp70‐x and PfHsp70‐x_T_ to suppress the aggregation of MDH and luciferase was also investigated, respectively (Figure 4C,D). The chaperones suppressed aggregation of luciferase/MDH in the same fashion both in the absence or presence of ADP. This was consistent with our previous findings that PfHsp70‐x and PfHsp70‐x_T_ assume similar conformation both in the absence of nucleotide or presence of ADP (Figure 1C). On the other hand, as previously observed for PfHsp70‐1,^22^ PfHsp70‐x/PfHsp70‐x_T_ demonstrated less protein aggregation suppression function in the presence of ATP (Figure 4C,D). Altogether, our findings demonstrate that PfHsp70‐x exhibits holdase chaperone function (binds misfolding protein to inhibit aggregation) and that removal of the C‐terminal EEVN motif marginally compromises the molecular chaperone's function.

### 3.5 PfHsp70‐x demonstrates propensity to bind asparagine‐rich peptides

Hsp70 is known to bind to extended hydrophobic patches of peptides made up of 7 amino acids.^26^ The *P. falciparum* proteome is characterized by at least one quarter of aggregation‐prone, asparagine repeats rich proteins.^20^ For this reason, we sought to establish the substrate preferences of PfHsp70‐x as such information could shed light on its role both in the trafficking and proteostasis of parasite proteins destined for the RBC. Thus we analyzed the comparative binding affinities of PfHsp70‐x/PfHsp70‐x_T_ for a battery of synthetic peptide substrates in our library (Supporting Information Table S1) using SPR analysis. The peptide represented by the sequence, NRLLTG was used as a model substrate of canonical Hsp70 (*E. coli* DnaK^26^). The second peptide, ALLLMYRR was derived from chicken mitochondrial aspartate amino‐transferase,^29^ while the third peptide GFRVVLMYRF, was derived from amino acids 256‐268 of *firefly* luciferase.^30^ To investigate if PfHsp70‐x demonstrates propensity to bind peptides enriched with asparagine residues, each of the three above mentioned peptides was modified by substituting the three middle residues with asparagine residues (Table 3). Both PfHsp70‐x and PfHsp70‐x_T_ bound to the peptide, NRLLTG and GFTVVLMYRF in comparable orders of affinities. Interestingly, both PfHsp70‐x and PfHsp70‐x_T_ bound to peptides NR**NN**TG and GFT**NNN**MYRF (asparagine enriched forms) with higher affinity than they had for the original peptides, respectively (Table 3). This suggests that PfHsp70‐x exhibits preference for asparagine repeat rich peptides. PfHsp70‐x bound to the peptide, ALLLMYRR within nanomolar range only in the absence of nucleotide or presence of ADP. Altogether, PfHsp70‐x exhibited the least affinity for this peptide than it had for the other two peptides. However, the asparagine enriched peptide, A**NNN**MYRR, bound to PfHsp70‐x with much higher affinity than the original peptide (ALLLMYRR), further supporting that the presence of asparagine residues in the peptides promote their recognition by PfHsp70‐x. However, PfHsp70‐x lacking the C‐terminal residues, EEVN had insignificant affinity for the peptide, ALLLMYRR. Insertion of asparagine residues to the peptide, ALLLMYRR did not significantly improve PfHsp70‐x_T_'s affinity for this peptide.

**Table 3:** Binding affinities of PfHsp70‐x/PfHsp70‐x_T_ for peptide substrates

	PfHsp70‐x *K*_D_ (M) [±SD]			PfHsp70‐x_T_ *K*_D_ (M) [±SD]		
Peptide sequence	NN	ATP	ADP	NN	ATP	ADP
NRLLTG	2.50 (± 0.34) e^−7^	1.70 (± 0.30) e^−6^	6.20 (± 0.12) e^−7^	2.80 (± 0.14) e^−7^	1.80 (± 0.23) e^−6^	1.70 (± 0.34) e^−7^
NR**NN**TG	6.91 (± ± 0.43) e^−9^	1.37 (± ± 0.20) e^−7^	4.31 (± ± 0.30) e^−9^	7.92 (± 0.50) e^−9^	1.07 (± 0.20) e^−7^	1.69 (± 0.30) e^−9^
ALLLMYRR	1.47 (±0.30) e^−6^	6.06 (±0.0.41) e^−5^	2.82 (±0.16) e^−6^	5.42 (0.33) e^−4^	ND	3.13 (±0.42) e^−4^
A**NNN**MYRR	2.21 (±0.53) e^−9^	7.83 (±0.33) e^−7^	2.21 (±0.31) e^−9^	6.26 (±0.32) e^−4^	ND	4.43 (±0.43) e^−4^
GFTVVLMYRF	1.09 (± 0.10) e^−7^	1.90 (± 0.12) e^−6^	2.85 (± 0.20) e^−7^	2.99 (± 0.10) e^−7^	1.10 (± 0.20) e^−6^	1.76 (±0.11) e^−7^
GFT**NNN**MYRF	2.40 (± 0.15) e^−9^	3.97 (±0.15) e^−7^	6.25 (± 0.80) e^−9^	2.20 (±0.12) e^−9^	5.17 (± 0.16) e^−7^	1.40 (± 0.15) e^−9^

ND, affinity too low to be determined; in bold are N residue insertions introduced in each peptide. The association rate constant represented by (*k*_a_), dissociation rate constants (*k*_d_), were used to determine the equilibrium constant (denotes the affinity) represented by (*K*_D_). SDs about the respective means are shown.

It is known that in general, nucleotide free‐ and ADP‐bound forms of Hsp70 bind to peptide substrates with comparable affinity.^22^ As expected, nucleotide free‐ and ADP‐bound forms of PfHsp70‐x/PfHsp70‐x_T_ bound to the various peptide substrates with affinities of similar magnitudes (Table 3). On the other hand, in the presence of ATP, binding affinity for the peptides by both PfHsp70‐x and PfHsp70‐x_T_ was reduced. Altogether, our findings suggest that PfHsp70‐x demonstrates affinity for peptides enriched with asparagine. Furthermore, the C‐terminal EEVN motif is not important for the recognition of peptides that exhibit high affinity for PfHsp70‐x. However, the presence of the EEVN motif enhances the affinity of PfHsp70‐x for peptides that bind to the chaperone with low affinity.

### 3.6 PfHsp70‐x interacts with human hop in vitro

PfHsp70‐x and hHop are reported to localize in the RBC cytosol.^12^, ^13^, ^14^, ^15^, ^16^, ^17^ Human Hsp90 has also been reported to be present in the RBC.^17^ Notably, PfHsp70‐x possesses the C‐terminal EEVN motif synonymous with the EEVD motif present in cytosolic Hsp70s of eukaryotic origin.^31^ The EEVD motif present in cytosolic Hsp70s is known to be crucial for binding to co‐chaperones, including Hop.^31^, ^32^ For this reason, Hsp70s with EEVD motifs normally cooperate with Hsp90 to facilitate‐fold of some proteins that require both Hsp70 and Hsp90 for complete folding. We therefore sought to enquire if PfHsp70‐x is capable of directly binding human Hop. It is plausible that if the PfHsp70‐x and hHop interact, their association could potentially bring both PfHsp70‐x and human Hsp90 into a heterologous protein folding complex between these chaperones of parasitic and human origin.

Using recombinant protein form, we investigated the direct association between PfHsp70‐x/PfHsp70‐x_T_ and hHop using the slot blot assay. The bait protein (hHop), negative control (BSA), and positive control (PfHsp70‐x) were immobilized onto a nitrocellulose membrane. Immunoblotting was carried out using α‐PfHsp70‐x antibody.^8^ The α‐PfHsp70‐x antibody was specific for PfHsp70‐x and exhibited no cross‐reactivity with either BSA or hHop (Supporting Information Figure 2A,B). The association of the recombinant proteins was investigated by overlaying the membrane with the prey protein (PfHsp70‐x/PfHsp70‐x_T_). Western blot analysis was conducted to detect if the PfHsp70‐x/PfHsp70‐x_T_ associated with the immobilized hHop. The association between PfHsp70‐x and human Hop was observed to occur in a concentration‐dependent manner (Supporting Information Figure 2C,D). This suggests that the observed association was specific. However, association between hHop and PfHsp70‐x_T_ was not observed (Supporting Information Figure 2C,D), suggesting that the EEVN motif is required for interaction between PfHsp70‐x and hHop to occur. The assay was repeated in the presence nucleotides 5 mM ATP/ADP. The addition of ADP appeared to enhance the association between hHop and PfHsp70‐x but the addition of ATP abrogated the interaction (Supporting Information Figure 2C,D). This is in line with a previous study which showed that PfHsp70‐1 and PfHop association was enhanced by ADP and in the presence of ATP the two proteins interacted with less affinity.^19^

**Figure 5:**
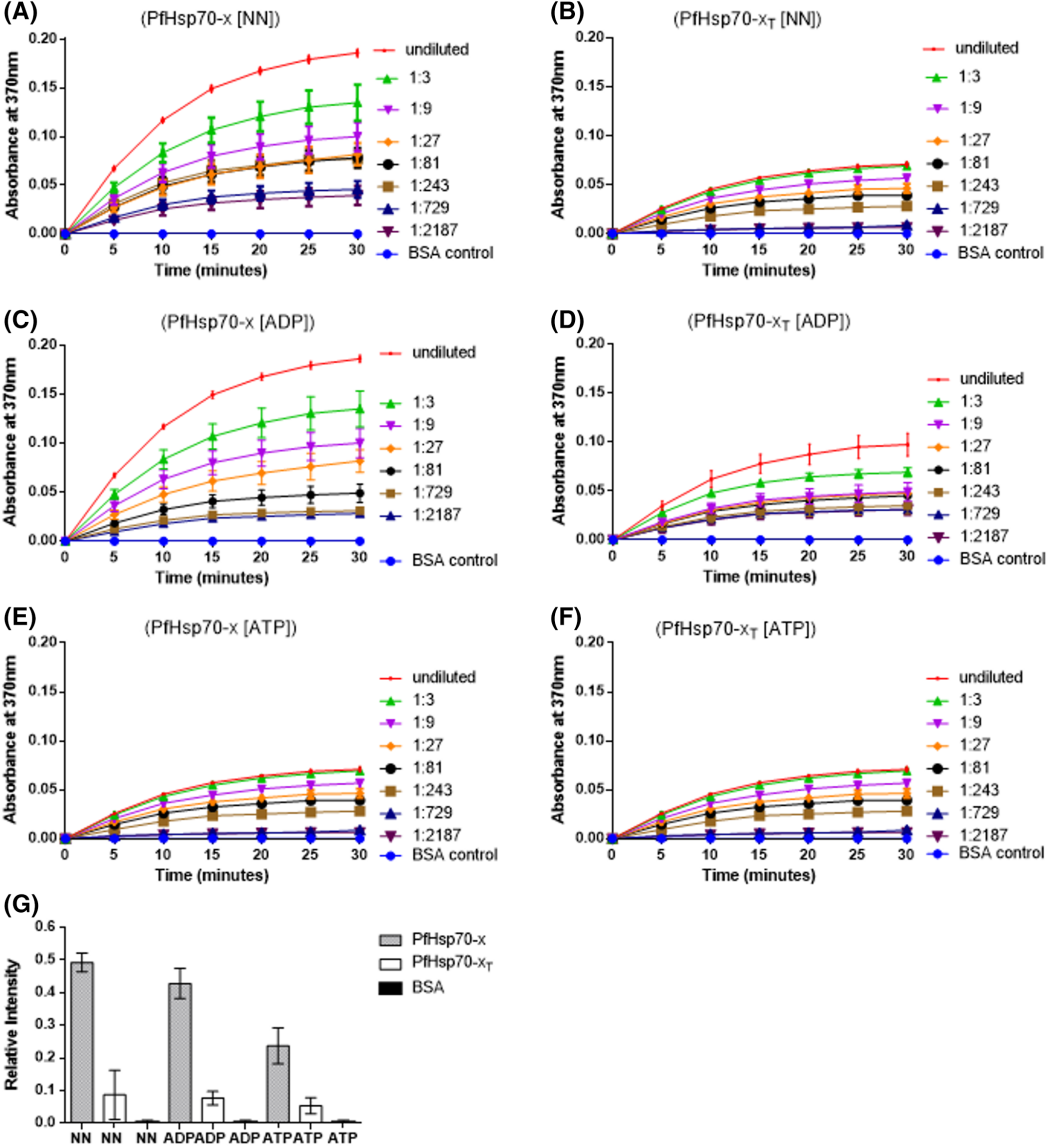
The EEVN motif of PfHsp70‐x is important for its interaction with human hop in vitro. The representative interaction curves generated from the ELISA analysis of PfHsp70‐x‐hHop association (A) and PfHsp70‐x_T_‐hHop association (B). The assay was conducted in the presence of varying amounts of hHop towards establishing dose‐dependent association. The assay was repeated in presence of 5 mM ADP for PfHsp70‐x‐hHop association (C) and PfHsp70‐x_T_‐hHop association (D); and in the presence of 5 mM ATP for PfHsp70‐x‐hHop association (E) and PfHsp70‐x_T_‐hHop association (F), respectively. The comparison of the relative binding affinities between PfHsp70‐x vs hHop, PfHsp70‐x_T_ versus hHop under different conditions were normalized to the maximum absorbance value obtained at the highest concentration of analyte used (G). The error bars represent the SDs obtained about the means of at least three assays conducted using independently purified recombinant protein batches [Color figure can be viewed at http://wileyonlinelibrary.com]

To confirm the direct interaction of PfHsp70‐x with hHop in vitro, an ELISA assay was conducted. PfHsp70‐x/PfHsp70‐x_T_ were immobilized onto the wells, while hHop or BSA (control) were used as analytes (Figure 5). A preliminary step consisted of demonstrating concentration dependent association between hHop and PfHsp70‐x (Figure 5A,B). The experiment was repeated in the presence of 5 mM ATP/ADP (Figure 5C‐F), respectively. The ELISA generated data shows that the association between PfHsp70‐x and hHop occurs in a concentration dependent form both in the absence of nucleotide or in the presence of ADP. On the other hand, ATP inhibited the association. The ELISA generated data further supported that the EEVN motif is required for interaction between PfHsp70‐x and hHop (Figure 5G).

**Figure 6:**
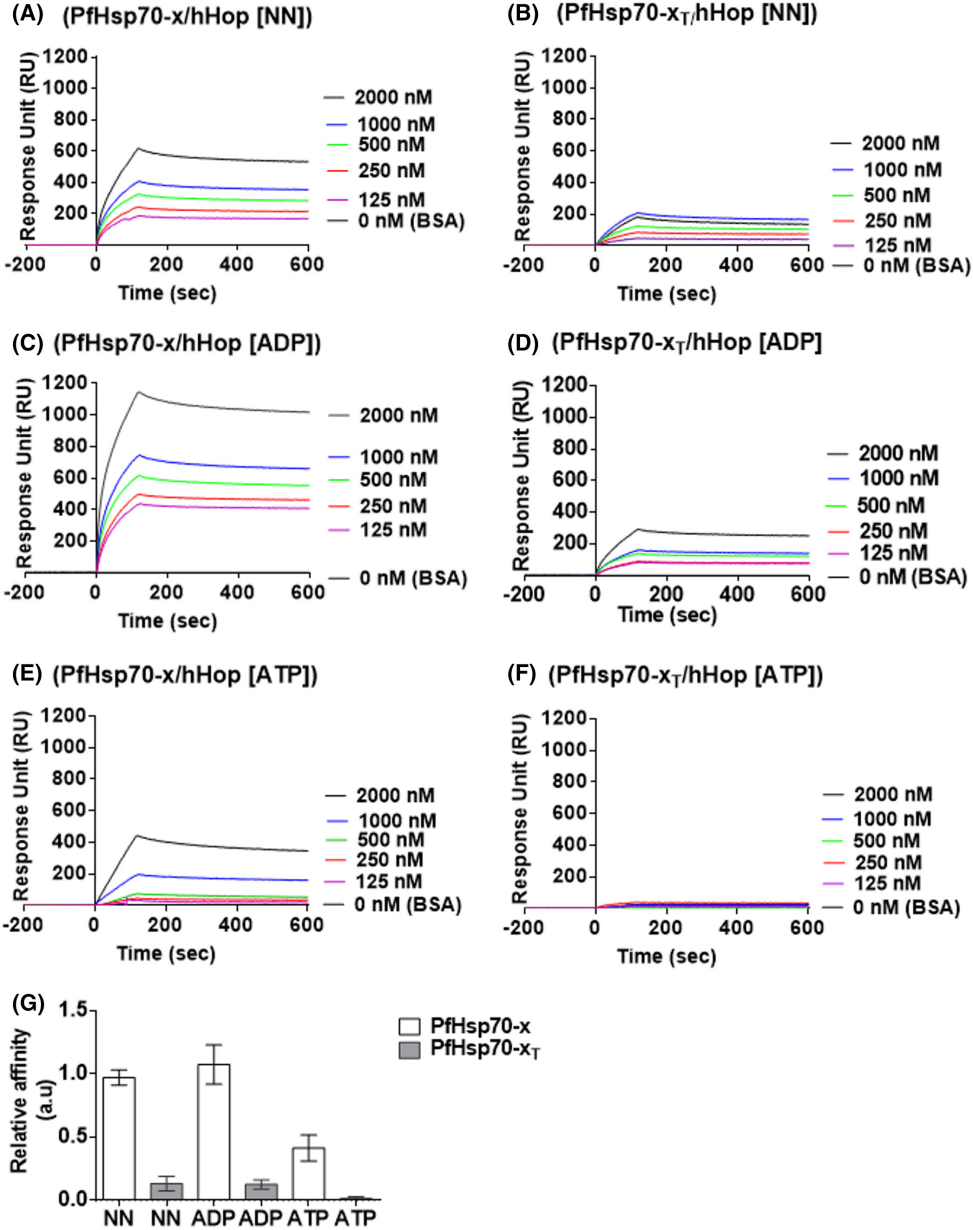
SPR analysis to confirm the direct interaction between PfHsp70‐x and human hop in vitro. The representative sensograms generated from the SPR analysis for PfHsp70‐x‐hHop association (A) and PfHsp70‐x_T_‐hHop association (B). The assay was conducted in the presence of varying amounts of hHop towards establishing dose‐dependent association. The assay was repeated in presence of 5 mM ADP for PfHsp70‐x‐hHop association (C) and PfHsp70‐x_T_‐hHop association (D); and in the presence of 5 mM ATP for PfHsp70‐x‐hHop association (E) and PfHsp70‐x_T_‐hHop association (F), respectively. The relative affinities for the association of PfHsp70‐x‐hHop and PfHsp70‐x_T_‐hHop under different conditions were normalized to the kinetics of PfHsp70‐x‐hHop in the absence of nucleotides (G). There was significant difference in the respective affinities of hHop for PfHsp70‐x compared to PfHsp70‐x_T_ (*P* < .05). The error bars represent the standard deviations obtained about the means of at least three assays using independently purified recombinant protein batches [Color figure can be viewed at http://wileyonlinelibrary.com]

To further validate the association of PfHsp70‐x and hHop using the recombinant forms of the respective proteins, interaction kinetics and affinity analysis were conducted using SPR. PfHsp70‐x/PfHsp70‐x_T_ were immobilized onto the chip while hHop or BSA (control) were used as analytes (Figure 6). The experiment was conducted in the absence of nucleotides (Figure 6A,B) and repeated in the presence of 5 mM ADP/ATP (Figure 6C‐F), respectively. As observed using both the slot blot assay and ELISA analysis, SPR generated data confirmed that the association between PfHsp70‐x and hHop occurs with comparable affinities in the absence of nucleotide or in the presence of ADP (Figure 6C,D). On the contrary, ATP inhibited the association by at least tenfold (Table 4). The SPR generated data further supported that the EEVN motif is required for interaction between PfHsp70‐x and hHop (Figure 6; Table 4).

**Table 4:** Binding kinetics of PfHsp70‐x and human hop

Ligand	Analyte	*k*_a_ (1/Ms)	*k*_d_ (1/s)	*K*_D_ (M)	*X* ^2^
PfHsp70‐x	hHop	4.30 (± 0.40) e^3^	2.30 (± 0.35) e^−5^	3.01 (± 0.15) e^−8^	1.1
	hHop +ADP	3.30 (± 0.12) e^3^	3.70 (± 0.43) e^−5^	2.31 (± 0.25) e^−8^	1.31
	hHop +ATP	3.47 (± 0.40) e^3^	4.40 (± 0.36) e^−4^	3.80 (± 0.35) e^−7^	1.5
PfHsp70‐x_T_	hHop	2.47 (± 0.14) e^1^	1.01 (± 0.22) e^−5^	2.10 (± 0.12) e^−6^	3.92
	hHop+ADP	4.26 (± 0.54) e^1^	2.10 (± 0.24) e^−5^	1.66 (± 0.05) e^−6^	1.09
	hHop +ATP	5.41 (± 0.10) e^1^	3.20 (± 0.14) e^−4^	4.10 (± 0.13) e^−5^	3.2

Table legends: ***k***_**a**_ is the association rate constant, ***k***_**d**_ dissociation rate constant, ***K***_**D**_ equilibrium constant, ***X*** ^**2**^ is the chi‐squared value correlation for SPR sensorgram fitting to the Langmuir model. Data is presented as mean plus/minus SD.

## 4 DISCUSSION

Although PfHsp70‐x is not essential for parasite survival,^9^, ^10^ a recent study by Charnaud et al.^9^ suggested that it is implicated in host cell invasion and immune evasion. Central to the development of clinical malaria pathology, is the export of the parasite protein, erythrocyte membrane protein 1 (PfEMP1) to the RBC surface. Parasite proteins that are destined for the infected RBC surface facilitate the development of malaria pathology by remodeling the parasite‐infected host RBCs to make them cyto‐adherent. The role of PfHsp70‐x in regulating cyto‐adherence of parasite‐infected RBCs is thought to be through its possible role in facilitating export of parasite proteins destined for the RBC.^8^, ^13^ In addition, PfHsp70‐x is also implicated in the folding of proteins of parasite origin that are exported to the host RBCs.^8^ For this reason, although PfHsp70‐x is not essential for parasite survival,^9^, ^10^ its implication in the invasion of the host and host immune evasion^10^, ^13^ makes it an important protein.

Here, we expressed and purified recombinant PfHsp70‐x (full length) protein and a truncated version of the protein with a missing C‐terminal EEVN motif. We established that PfHsp70‐x exhibits preference for asparagine repeat rich peptides. Nearly a quarter of malarial proteins are asparagine repeat rich.^20^ Thus, our data suggest that PfHsp70‐x may preferentially bind to malarial proteins that are exported to the parasite‐infected RBC. We further established that PfHsp70‐x directly binds to human Hop protein in vitro. This suggests that human Hop may modulate the possible functional cooperation between PfHsp70‐x and human Hsp90 chaperone, which is known to co‐localize with PfHsp70‐x in the RBC. However, it remains to be validated if PfHsp70‐x does associate with human Hop in vivo.

In general, the SBD of Hsp70 is subdivided into a β‐subdomain that is approximately 15 kDa in size and an α‐helical lid subdomain with an average size of 10 kDa that is located at the C‐terminus. The β‐subdomain makes direct contact with the substrate while the lid segment is thought to clamp the substrate bound by the β‐subdomain of Hsp70.^31^ Cytosolic Hsp70s of eukaryotic origin possess a C‐terminal motif characterized by residues, EEVD. The EEVD motif is thought to interact with co‐chaperones such as Hsp40 and Hop.^31^, ^32^ In addition, the EEVD motif of Hsp70 is implicated both in the substrate binding and ATPase activities of Hsp70.^32^ Whereas PfHsp70‐1 (cytosolic homologue) of *P. falciparum* possesses EEVD residues at its C‐terminus, the parasite exported form, PfHsp70‐x, possesses residues EEVN at the C‐terminus. Of the six, Hsp70s expressed by the parasite, PfHsp70‐1 and PfHsp70‐x share the highest amino acid sequence identities.^3^ For this reason, it was initially thought that PfHsp70‐x was resident in the parasite cytosol.^3^ Thus, the high sequence identity between PfHsp70‐1 and PfHsp70‐x suggests that PfHsp70‐x most probably binds to peptide substrates that are similar to those that are recognized by PfHsp70‐1. To understand the role of PfHsp70‐x, we sought to establish its substrate preferences. Our findings demonstrated that PfHsp70‐x preferentially binds asparagine repeat rich peptides (Table 3). The removal of the EEVN motif from PfHsp70‐x did not affect the peptide binding affinity of PfHsp70‐x for peptides, NRLLTG, and GFRVVLMYRF and their asparagine enriched equivalents (Table 3). On the other hand, the absence of the EEVN motif from PfHsp70‐x resulted in the reduction of the affinity of the chaperone for the aspartame amino transferase derived peptide, ALLLMYRR (Table 3). Interestingly, enriching the peptide, ALLLMYRR with asparagine residues gave rise to a drastic increase in binding affinity by full length PfHsp70‐x protein (Table 3). However, the presence of the asparagine residues in the peptide, ALLLMYRR did not improve the binding affinity of the EEVN truncated mutant (PfHsp70‐x_T_) for this peptide (Table 3) under all the experimental conditions (presence of ADP/ADP or absence of the nucleotides). Altogether, our findings suggest that PfHsp70‐x demonstrates affinity for peptides enriched with asparagine and that the EEVN motif though not required for binding all peptides, is important for the recognition of some peptides.

We further established that the EEVN motif of PfHsp70‐x is important for the conformational integrity of the protein. This is in line with a previous study which suggested that mutations in the EEVD motif of eukaryotic Hsp70 affects the global conformation of the protein.^32^ It has previously been demonstrated that PfHsp70‐x is capable of suppressing the heat‐induced aggregation of protein in vitro.^33^ Here, we further confirmed this by demonstrating that PfHsp70‐x was capable of suppressing heat‐induced aggregation of MDH and luciferase in vitro (Figure 4). Furthermore, we established that PfHsp70‐x deficient of the EEVN residues was less effective at suppressing the heat‐induced aggregation of MDH and luciferase. This is in agreement with a previous study that suggested that the EEVD motif of cytosolic Hsp70 is important for the substrate refolding function of the chaperone.^34^ However, it remains unclear why PfHsp70‐x possesses an asparagine (N) residue at the C‐terminus instead of aspartic acid (D) which is present in most eukaryotic cytosolic Hsp70 homologues such as PfHsp70‐1.

In cytosolic eukaryotic Hsp70s, the C‐terminal EEVD residues are thought to facilitate interaction of the C‐terminus and the N‐terminus.^32^, ^34^ This probably explains why deletion of the EEVN motif of PfHsp70‐x resulted in at least 100‐fold reduced affinity for ATP (Table 1). Indeed, the EEVN minus form of PfHsp70‐x also exhibited reduced ATP hydrolyzing capability (Figure 3; Table 2). It has been demonstrated that mutations targeting the EEVD motif of Hsp70 result in reduced ATPase activity of the protein.^32^ Thus, our findings implicate the EEVN motif of PfHsp70‐x both in the binding of ATP and in its subsequent hydrolysis.

There is possibility that PfHsp70‐x may cooperate with human Hsp90 via a human Hop‐mediated partnership. Therefore, in the current study we sought to establish if PfHsp70‐x has capability to recognize human Hop. Interestingly, our data suggest that PfHsp70‐x associates with human Hop in the nanomolar range (Table 4). Furthermore, the two proteins associate with less affinity in the presence of ATP compared to conditions in which there is no nucleotide or presence of ADP. This is in line with a previous observation we made that the parasite cytosolic protein, PfHsp70‐1, interacted with PfHop with higher affinity in the presence of ADP/absence of nucleotide compared to a scenario in which ATP was present.^5^, ^19^ The absence of the EEVN residues reduced the affinity of PfHsp70‐x for human Hop. The EEVD motif of cytosolic Hsp70s is known to facilitate interaction of the chaperone with its co‐chaperone partners such as Hsp40 and Hop.^30^, ^31^ Thus, our findings suggest a similar role for the EEVN residues of PfHsp70‐x in mediating its possible functional association with human Hop. However, it remains to be validated if PfHsp70‐x does occur in complex with human Hop resident in the parasite‐infected RBC.

This study also investigated the secondary structure and thermal stability of PfHsp70‐x. The far‐UV spectra exhibited a positive peak centered at 194 nm and two troughs at 208 and 223 nm, respectively (Figure 1). This spectrum represents the predominantly α‐helical character of the recombinant protein. Furthermore, PfHsp70‐x displayed resilience to thermal stress as 50% of the recombinant protein remained folded at 70°C (Figure 1B). We have previously observed that the *P. falciparum* cytosolic Hsp70s (PfHsp70‐1 and PfHsp70‐z) both exhibit stability at fairly high temperatures.^21^, ^22^, ^24^ Heat shock proteins maintain other proteins in a competent folding state under physiologically stressful conditions. For this reason, they need to be stable under stressful conditions in order to maintain proteostasis under such conditions. This is especially important for survival of malaria parasites during clinical malaria progression as the disease is characterized by periodic fever conditions which inherently challenge the parasite's proteomic integrity. Incidentally, it is thought that the development of malaria fever is associated with development of knobs on the surface of the parasite‐infected RBCs. It is thought that the upregulation of parasite heat shock proteins is linked to increase in parasite circulation counts, thereby augmenting clinical malaria progression.^35^ Similarly, a recent study,^9^ proposed a role for PfHsp70‐x in enhancing parasite infectivity. Thus, the stability of PfHsp70‐x to heat stress represents an important attribute for the parasite at the clinical phase of malaria.

Altogether, our findings established that the C‐terminal EEVN motif of PfHsp70‐x is important for the conformational integrity of the protein. In addition, deletion of the EEVN residues results in a protein whose ATP binding, ATP hydrolysis and chaperone functions are compromised. In addition, we demonstrated that PfHsp70‐x exhibits propensity to bind asparagine repeat rich peptides. Furthermore, the EEVN motif enhances the affinity of PfHsp70‐x for peptides substrates. The preference of PfHsp70‐x for asparagine repeat rich substrates suggests that this molecular chaperone may possibly bind unstable proteins of parasitic origin to facilitate their trafficking to the RBC. As PfHsp70‐x occurs in the PV and the RBC,^8^ its presence in the RBC may be important for the folding of protein cargo that is trafficked from the parasite. In addition, we established that PfHsp70‐x interacts with human Hop in vitro. As part of future studies, we intend to establish if PfHsp70‐x occurs in a functional complex with human Hsp90 via a human Hop mediated partnership. Should such a functional partnership exists it would suggest a role for PfHsp70‐x in folding of some proteins resident in the parasite‐infected RBCs that possibly require both PfHsp70‐x and human Hsp90 for their complete folding. Indeed, there is growing evidence that chaperones and co‐chaperones of parasite and human origin may cooperate in the in the infected red blood cell to facilitate folding of exported parasite proteins.^35^ This has raised prospects of developing antimalarial chemotherapeutic agents targeting chaperone networks.^35^, ^36^, ^37^, ^38^

## Funding information

Deutsche Forchungsgemeinshaft (DFG), Grant number: L1/402/14‐1; Department of Science and Technology/National Research Foundation (NRF) of South Africa, Grant numbers: UID, 75464, UID, 92598; South African Research Chairs Initiative of the Department of Science and Technology and National Research Foundation, Grant number: 64788; National Research Foundation Masters Scholarship; South African National Research Foundation Post‐Doctoral Fellowship, Grant number: UID, 111989; African‐German Network of Excellence in Science Junior Researcher Grant; Georg Foster Research Fellowship Awarded by the Alexander von Humboldt Foundation, Germany.

## Supporting information

Supplementary Figure 1 Expression and purification of recombinant proteins
**Supplementary Figure 2** PfHsp70‐x directly interacts with human Hop

Supplementary Table 1 Binding affinities of PfHsp70‐x/PfHsp70‐xT for peptide substrates
