# Supplementary material for: Structural and biochemical characterization of Plasmodium falciparum Hsp70‐x reveals functional versatility of its C‐terminal EEVN motif
Source: Proteins. 2018 Sep 29;86(11):1189–201. doi: 10.1002/prot.25600 (PMC6282620; doi:10.1002/prot.25600)
Supplement: Supplementary file 1 — Supplementary Figure 1 Expression and purification of recombinant proteins Supplementary Figure 2 PfHsp70‐x directly interacts with human Hop [file PROT-86-1189-s001.pdf]

Supplementary Figure 1

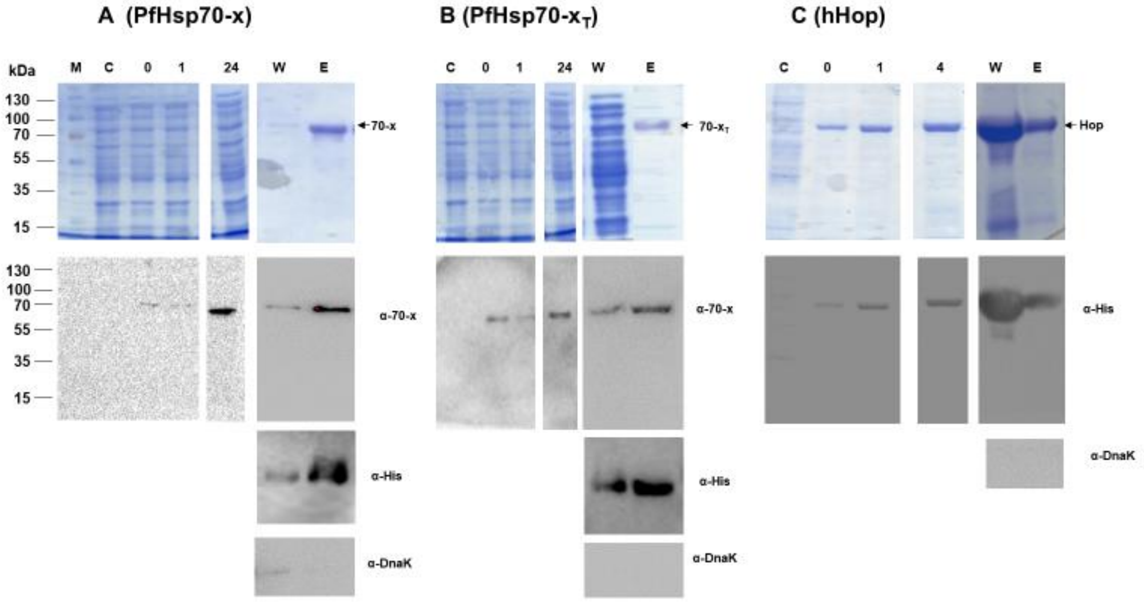

Expression and purification of recombinant proteins

(A) PfHsp70-x, (B) PfHsp70-x_T and (C) hHop were expressed in *E. coli* XL1 Blue cells transformed with pQE30/PfHsp70-x/PfHsp70-x_T and pQE30/hHop. SDS-PAGE (12 %) and Western analyses (lower panel) for the expression of recombinant PfHsp70-x/PfHsp70-x_T/hHop, the purification of the recombinant proteins was conducted as previously described (19; 20). Lane M – Page ruler (Thermo Scientific) in kDa is shown on the left hand side; lane C - the total extract for cells transformed with a neat pQE30 plasmid; lane 0 – total cell extract transformed prior to IPTG induction; lanes 1, 4, 24 – total cell lysate obtained 1, 4 hr and overnight post induction. Lane W; washes, and lane E—recombinant protein eluted using 500 mM imidazole. Lower panels: Western blot confirming expression, purification of PfHsp70-x/PfHsp70-x_T and hHop. The Western blots were based on α-PfHsp70-x and α-His used as primary antibodies, respectively. Furthermore, to confirm the purity of PfHsp70-x/PfHsp70-x_T and hHop from DnaK contamination, Western blotting was conducted using α-DnaK antibodies.

Supplementary Figure 2

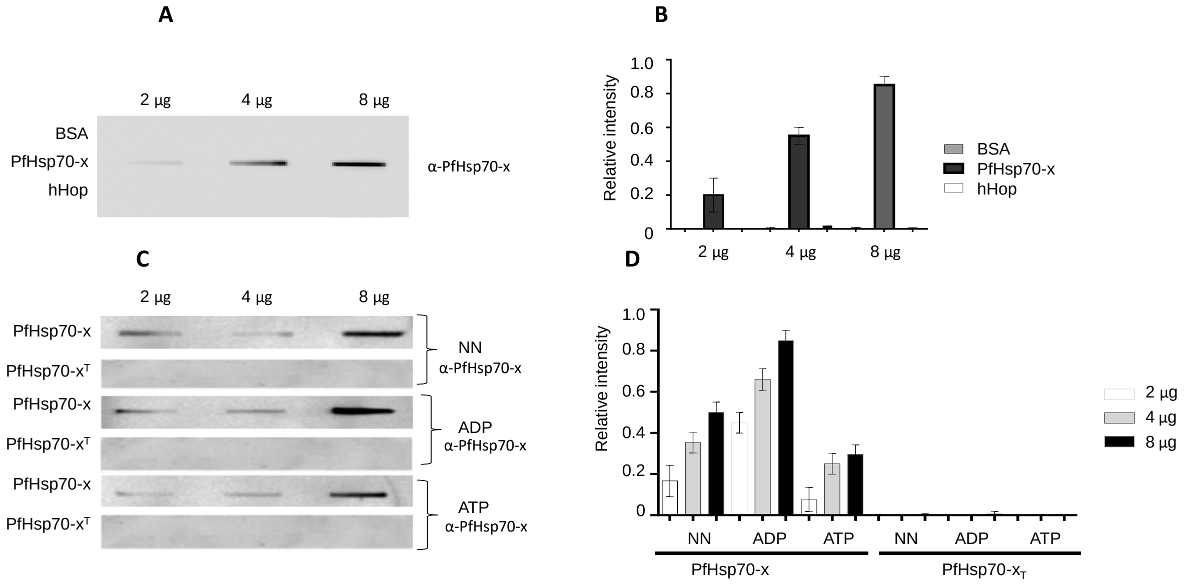

PfHsp70-x directly interacts with human Hop

Slot blot analysis (**A**) and accompanying densitometric readings (**B**) confirming the specificity of α-PfHsp70-x antibodies were first conducted; Subsequent to this, slot blot analyses (**C**) and accompanying densitometric readings (**D**) to demonstrate interaction of PfHsp70-x/PfHsp70-x_T and human Hop were conducted. The assays were conducted either in the absence of nucleotide (NN); or presence of 5 mM ADP/ATP, respectively. The data was obtained from at least three independent assays conducted using different protein batches. Error bars are indicated. The association between PfHsp70-x and hHop was demonstrated to be dose dependent and was further ascertained by densitometric analysis; ANOVA ($p<0.001$).
