# Supplementary material for: Structural and biochemical characterization of Plasmodium falciparum Hsp70‐x reveals functional versatility of its C‐terminal EEVN motif
Source: Proteins. 2018 Sep 29;86(11):1189–201. doi: 10.1002/prot.25600 (PMC6282620; doi:10.1002/prot.25600)
Supplement: Supplementary file 2 — Supplementary Table 1 Binding affinities of PfHsp70‐x/PfHsp70‐xT for peptide substrates [file PROT-86-1189-s002.pdf]

Supplementary Table 1. Binding affinities of PfHsp70-x/PfHsp70-x_T for peptide substrates

Ligand	Analyte	k_a (1/ Ms)	k_d (1/s)	K_D (M)	χ2
PfHsp70-x	-	NS	NS	NS	ND
	NRLLTG	3.86 (± 0.20)	9.97 (± 0.41) e^{-6}	2.50 (± 0.34) e^{-7}	0.98
	NRLLTG + ADP	1.62 (± 0. 22)	1.01 (± 0.28) e^{-5}	6.20 (± 0.12) e^{-7}	4.38
	NRLLTG + ATP	1.21 (± 0.23)	1.01 (± 0.13) e^{-5}	1.70 (± 0.30) e^{-6}	2.10
PfHsp70-x_T	-	NS	NS	NS	ND
	NRLLTG	3.15 (± 0.31)	8.67 (± 0.42) e^{-6}	2.80 (± 0.14) e^{-7}	0.24
	NRLLTG + ADP	6.0 (± 0.51)	1.00 (± 0.21) e^{-5}	1.70 (± 0.34) e^{-7}	3.21
	NRLLTG + ATP	3.18 (± 0.42) e^{1}	1.61(± 0.36) e^{-4}	1.80 (± 0.23) e^{-6}	2.37
PfHsp70-x	NR**NN**TG	2.43 (± 0.45)	9.49 (± 0.27) e^{-7}	6.91 (± 0.43) e^{-9}	1.31
	NR**NN**TG +ADP	2.55 (± 0.23)	1.03 (± 0.41) e^{-7}	4.31 (± 0.30) e^{-9}	3.98
	NR**NN**TG +ATP	7.33 (± 0.22)	1.01 (± 0.31) e^{-6}	1.37 (± 0.20) e^{-7}	4.10
PfHsp70-x_T	NR**NN**TG	3.52 (± 0.18)	2.79 (± 0.19) e^{-8}	7.92 (± 0.50) e^{-9}	1.56
	NR**NN**TG +ADP	5.96 (±0.1)	1.01 (± 0.31) e^{-8}	1.69 (± 0.30) e^{-9}	2.10
	NR**NN**TG +ATP	9.37 (±0.17)	1.01 (± 0.21) e^{-5}	1.07 (± 0.20) e^{-7}	3.91
PfHsp70-x	GFTVVLMYRF	4.03 (± 0.19) e^{2}	1.03 (± 0.40) e^{-5}	1.09 (± 0.10) e^{-7}	0.812
	GFTVVLMYRF + ADP	3.53 (± 0.18) e^{2}	1.00 (± 0.14) e^{-5}	2.85 (± 0.20) e^{-7}	2.18
	GFTVVLMYRF + ATP	1.11 (± 0.20) e^{2}	1.17 (± 0.44) e^{-4}	1.90 (± 0.12) e^{-6}	4.12

PfHsp70-x_T	GFTVVLMYRF	2.48 (± 0.40) e^{1}	2.52 (± 0.34) e^{-6}	2.99 (± 0.10) e^{-7}	0.68
	GFTVVLMYRF + ADP	1.14 (± 0.19) e^{2}	1.01 (± 0.22) e^{-5}	1.76 (±0.11) e^{-7}	2.36
	GFTVVLMYRF + ATP	1.02 (± 0.16) e^{2}	1.01 (± 0.23) e^{-4}	1.10 (± 0.20) e^{-6}	5.91
PfHsp70-x	GFT**NNN**MYRF	9.03 (± 0.34) e^{2}	1.28 (± 0.2) e^{-7}	2.40 (± 0.15) e^{-9}	0.89
	GFT**NNN**MYRF + ADP	4.60 (± 0.15) e^{2}	1.01(± 0.23) e^{-7}	6.25 (± 0.80) e^{-9}	4.21
	GFT**NNN**MYRF + ATP	2.54 (± 0.13) e^{2}	1.01 (± 0.11) e^{-5}	3.97 (±0.15) e^{-7}	2.58
PfHsp70-x_T	Peptide 2 GFT**NNN**MYRF	3.60 (± 0.34) e^{2}	1.01(± 0.14) e^{-7}	2.20 (±0.12) e^{-9}	1.37
	GFT**NNN**MYRF + ADP	5.32 (± 0.45) e^{2}	1.01 (± 0.40) e^{-7}	1.40 (± 0.15) e^{-9}	3.10
	GFT**NNN**MYRF + ATP	8.57 (± 0.14) e^{2}	1.00 (± 0.20) e^{-5}	5.17 (± 0.16) e^{-7}	1.87
PfHsp70-x	ALLLMYRR	8.67 (±0.21) e^{-1}	1.28 (±0.92) e^{-6}	1.47 (±0.30) e^{-6}	3.03
	ALLLMYRR +ATP	2.65 (±0.11) e^{-2}	1.60 (±0.29) e^{-6}	6.06 (±.0.41) e^{-5}	2.57
	ALLLMYRR +ADP	1.04 (± 0.21) e^{3}	2.82 (±0.33) e^{-3}	2.82(±0.16) e^{-6}	1.50
PfHsp70-x_T	ALLLMYRR	1.92 (±0.31) e^{-2}	1.04 (±0.45) e^{-5}	5.42 (0.33) e^{-4}	1.95
	ALLLMYRR +ATP	ND	ND	ND	ND
	ALLLMYRR +ADP	3.32 (±0.21) e^{-2}	1.04 (±0.25) e^{-5}	3.13(±0.42) e^{-4}	1.89
PfHsp70-x	A**NNN**MYRR	3.31 (±0.31) e^{5}	7.32 (±0.43) e^{-4}	2.21 (±0.53) e^{-9}	4.59
	A**NNN**MYRR +ATP	7.24 (±0.34) e^{3}	5.67 (±0.11) e^{-3}	7.83 (±0.33) e^{-7}	3.78

	A**NNN**MYRR +ADP	1.74 (±0.13) e^{6}	3.86 (±0.24) e^{-3}	2.21 (±0.31) e^{-9}	1.12
PfHsp70-x_T	A**NNN**MYRR	1.67 (±0.34) e^{-2}	1.04 (±0.51) e^{-5}	6.26 (±0.32) e^{-4}	3.24
	A**NNN**MYRR +ATP	ND	ND	ND	ND
	A**NNN**MYRR +ADP	2.35 (±0.37) e^{-2}	1.04 (±0.21) e^{-5}	4.43 (±0.43) e^{-4}	2.66

ND, affinity too low to be determined; NS, No signal was detected; As a control a channel without immobilized protein was used as a background control for referencing; in bold are **N** residue insertions introduced in each peptide. The association rate constant is represented by (*ka*), dissociation rate constants (*kd*), and the equilibrium constant (denotes the affinity) is represented by (K_D). Standard deviations about the respective means are shown.
